# Crystal structure and Hirshfeld surface analysis of 6,6′-dimethyl-2,2′-bi­pyridine-1,1′-diium tetra­chlorido­cobaltate(II)

**DOI:** 10.1107/S2056989024005152

**Published:** 2024-06-11

**Authors:** Sivaraman Jagadeesan, Swinton Darious Robert, Perumal Venkatesan, Rajamanikandan Sundararaj, Krishnan Soundararajan, Jeeva Jasmine Nithianantham

**Affiliations:** a Department of Chemistry, Srimad Andavan Arts and Science College (Autonomous), Tiruchirappalli-620005, Tamilnadu, India; b PG & Research Department of Chemistry, Bishop Heber College (Autonomous), Tiruchirappalli-620017, Tamilnadu, India; c Center for Drug Discovery, Karpagam Academy of Higher Education, Coimbatore, Tamilnadu, India; d Department of Chemistry, Periyar Maniammai Institute of Science and Technology, Vallam-613403, Thanjavur, Tamilnadu, India; e Molecular Biophysics Unit Indian Institute of Science, Bangalore,India; University of Aberdeen, United Kingdom

**Keywords:** cobalt complex, substituted bi­pyridine, Hirshfeld surface analysis, crystal structure

## Abstract

In the title mol­ecular salt, (C_12_H_14_N_2_)[CoCl_4_], the dihedral angle between the pyridine rings of the cation is 52.46 (9)° and the N—C—C—N torsion angle is −128.78 (14)°, indicating that the ring nitro­gen atoms are in *anti*-clinal conformation. The Cl—Co—Cl bond angles in the anion span the range 105.46 (3)–117.91 (2)°. In the extended structure, the cations and anions are linked by cation-to-anion N—H⋯Cl and C—H⋯Cl inter­actions, facilitating the formation of *R*^4^_4_(18) and *R*^4^_4_(20) ring motifs. Furthermore, the crystal structure features weak anion-to-cation Cl⋯π inter­actions [Cl⋯π = 3.4891 (12) and 3.5465 (12) Å]. Hirshfeld two-dimensional fingerprint plots revealed that the most significant inter­actions are Cl⋯H/H⋯Cl (45.5%), H⋯H (29.0%), Cl⋯C/C⋯Cl (7.8%), Cl⋯N/N⋯Cl (3.5%), Cl⋯Cl (1.4) and Co⋯H (1%) contacts.

## Abstract 2

The title salt features N—H⋯Cl and C—H⋯Cl cation-to-anion hydrogen bonds and complementary anion-to-cation Cl⋯π inter­actions.

## 1. Chemical context

In recent years, non-covalent inter­actions have played an important role in organic**–**inorganic hybrid materials that have attracted researchers because of their potential applications in catalysis, energy storage devices, luminescence, photography and drug delivery (Bringley *et al.*, 2005 ▸; Avila-Montiel *et al.*, 2020 ▸). Cobalt(II) halide compounds are used as metal catalysts in various organic transformations and possess important fluorescence and magnetic properties (Decaroli *et al.*, 2015 ▸). As part of our work in this area, we now describe the synthesis, structure and Hirshfeld surface analysis of the title salt, C_12_H_14_N_2_^2+^·[CoCl_4_]^2–^, (**I**).
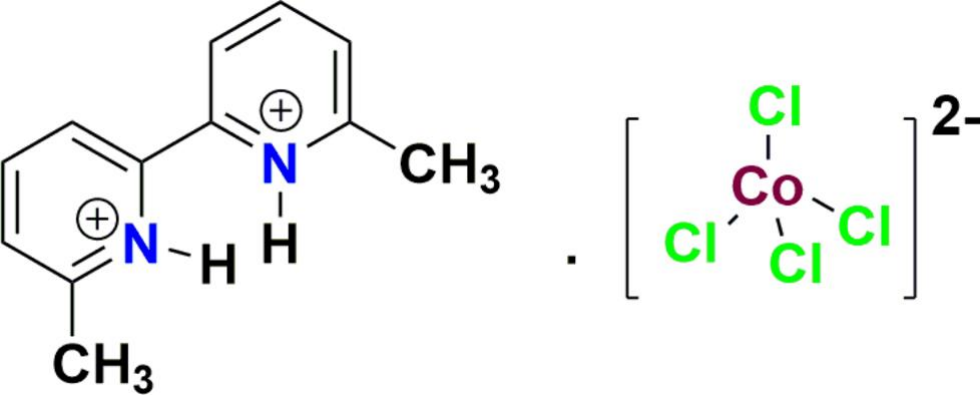


## 2. Structural commentary

**Figure 1:**
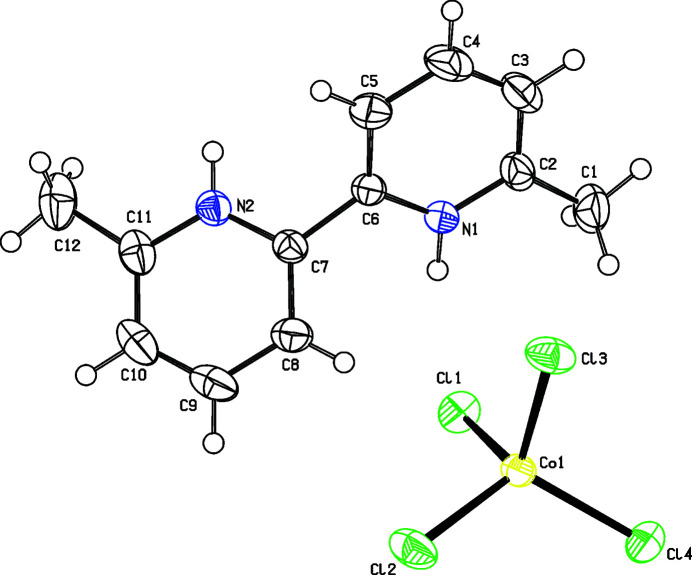
The mol­ecular structure of (**I**), with displacement ellipsoids drawn at the 50% probability level.

The asymmetric unit of (**I**) contains one C_12_H_14_N_2_^2+^ (DMB^2+^) cation and one [CoCl_4_]^2–^ anion in the triclinic space group *P*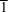 (Fig. 1 ▸). The pyridine ring nitro­gen atoms are protonated, which is confirmed by the widening of the C2—N1—C6 [123.47 (14)°] and C8—C7—N2 [119.92 (14)°] bond angles compared to a value of 118.4° in the neutral compound (Sengül *et al.*, 1998 ▸). The dihedral angle between the pyridine rings in (**I**) is 52.46 (9)°, showing that they are substanti­ally twisted with respect to each other. The values for the torsion angles C5—C6—C7—C8 [–127.95 (17)°] and N1—C6—C7—N2 [–128.78 (14)°] indicate that the nitro­gen atoms of the pyridine rings exhibit a (–)*anti*-clinal conformation. The Co—Cl bond lengths in the [CoCl_4_]^2–^ anion range from 2.2600 (6)–2.2997 (7) Å, where Cl1 and Cl4 have a longer distance than Cl2 and Cl3. The average Co—Cl bond length of 2.280 Å is consistent with that of similar complexes (Zhang *et al.*, 2005 ▸; Jebas & Balasubramanian, 2006 ▸). The Cl—Co—Cl bond angles are in the range 105.46 (3)–117.91 (2)° with an average bond angle of 111.13 (2)° (Azadbakht *et al.*, 2012 ▸; Mghandef & Boughzala, 2015 ▸). The smallest bond angle (Cl2—Co1—Cl3) correlates with the shortest Co—Cl bond lengths but there is no obvious correlation between bond lengths and the largest angle.

## 3. Supra­molecular features

**Figure 2:**
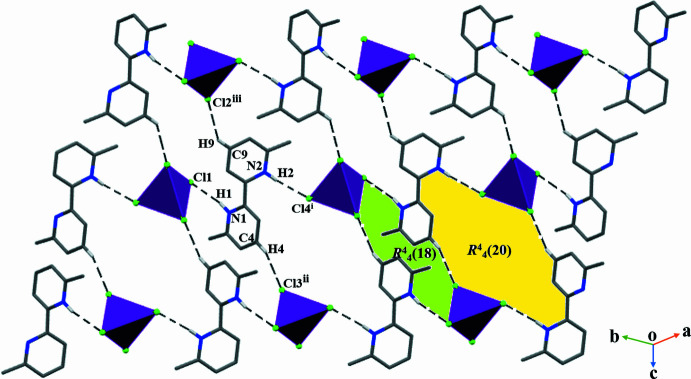
A view of the supra­molecular architecture of (**I**), showing the 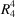
(18) and 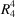
(20) loops. [Symmetry code: (iv) 1 + *x*, *y*, *z*.]

**Figure 3:**
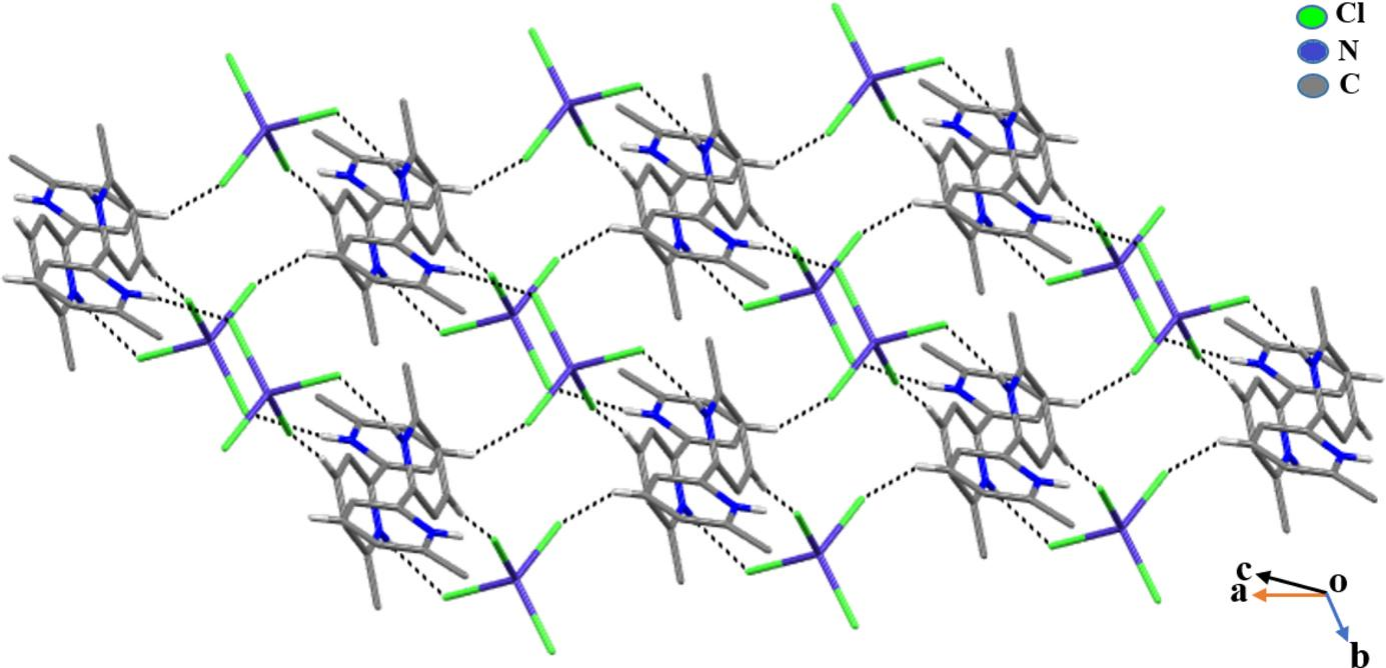
The wavy sheet-like two-dimensional supra­molecular architecture of (**I**) viewed along the *ab* direction. The black dotted lines represent hydrogen bonds.

In the extended structure of (**I**), the components are linked by N1—H1⋯Cl1 and N2—H2⋯Cl4 hydrogen bonds (Table 1 ▸), which generate infinite [1
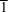
0] chains. Weak C4—H4⋯Cl3 and C9—H9⋯Cl2 hydrogen bonds also occur, so that each chlorine atom accepts one hydrogen bond. Together, the hydrogen bonds generate infinite sheets in which 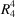
(18) and 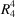
(20) loops are apparent (Fig. 2 ▸). A wavy sheet-like structure of the compound can be seen when the structure is viewed along the *ab*-axis direction (Fig. 3 ▸). The crystal structure also features weak anion⋯π inter­actions [Co1—Cl2⋯*Cg*2^iv^ = 3.5465 (12) Å; Co1—Cl3⋯*Cg*1^iv^ = 3.4891 (12) Å, where *Cg*1 is the centroid of the N1/C2–C6 ring and *Cg*2 is the centroid of the N2/C7–C11 ring; symmetry code: (iv) 1 + *x*, *y*, *z*] (Degtyarenko & Domasevitch, 2014 ▸).

**Figure 4:**
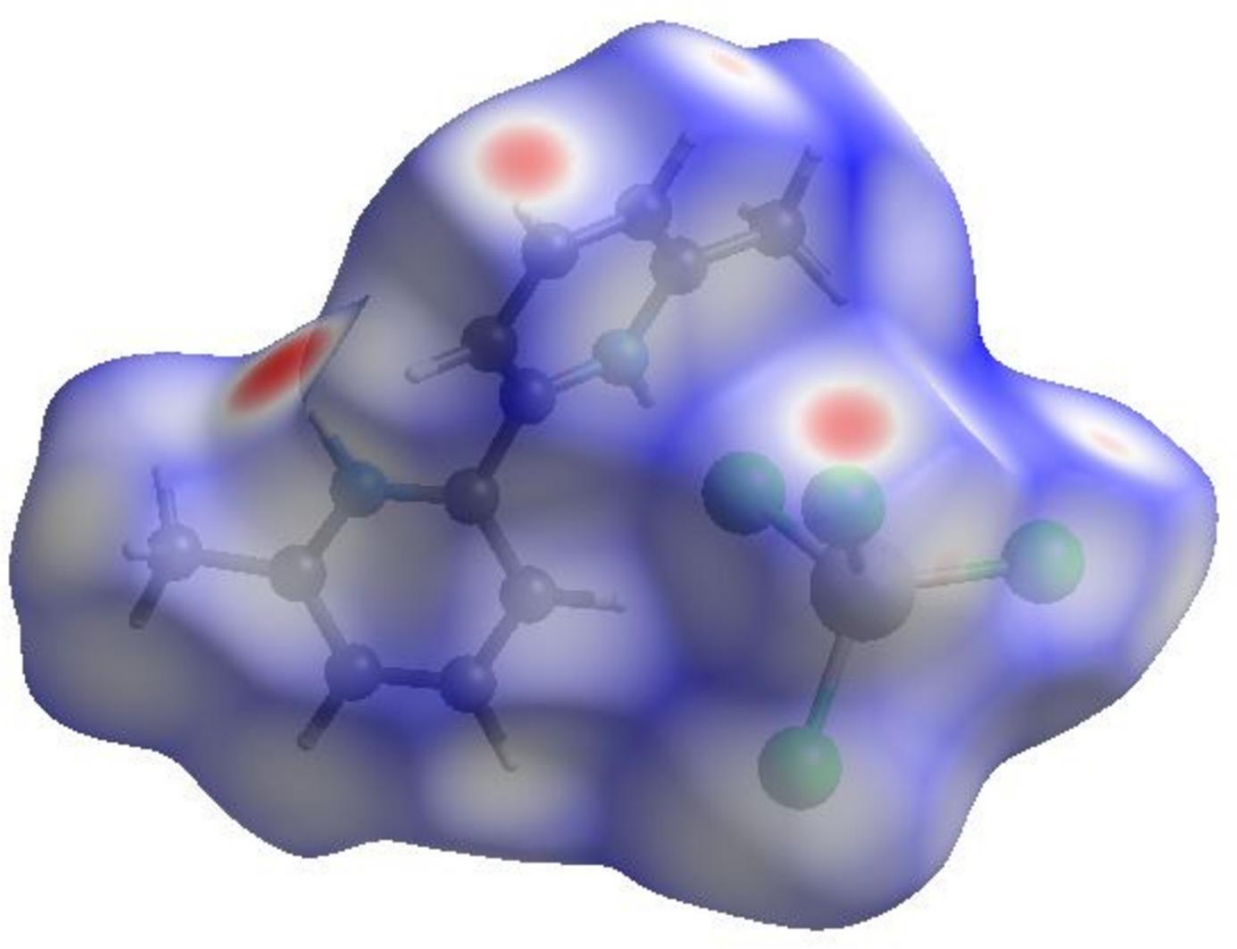
A view of the three-dimensional Hirshfeld surface of (I) mapped over *d*_norm_.

**Figure 5:**
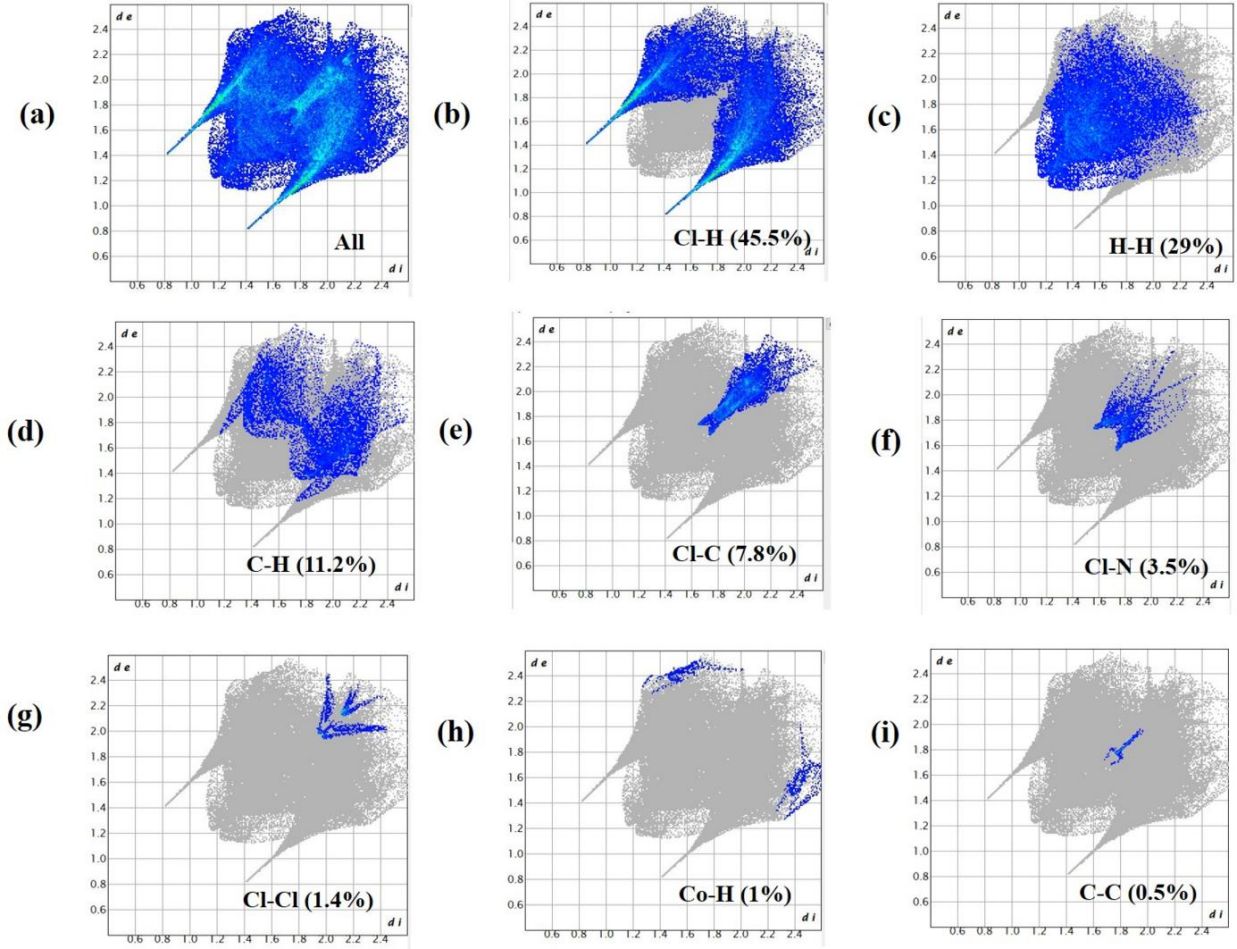
The two-dimensional fingerprint plot for (**I**) showing all inter­molecular inter­actions and delineated into Cl⋯H/H⋯Cl, H⋯H, C⋯H/H⋯C, Cl⋯C/C⋯Cl, Cl⋯N/N⋯Cl, Cl⋯Cl, Co⋯H and C⋯ contacts.

The Hirshfeld surface analysis and its related fingerprint plots were created with *Crystal Explorer 17.5* (Turner *et al.*, 2017 ▸). The Hirshfeld surface of the title salt (Fig. 4 ▸) mapped over *d*_norm_ within the range −0.43 to 1.17 a.u. shows bright red spots within the locales of *D*⋯*A* (*D* = donor, *A* = acceptor) inter­actions, as expected. The two-dimensional fingerprint plots (Fig. 5 ▸) show that the most significant contacts are Cl⋯H/H⋯Cl (45.5%), H⋯H (29.0%), C⋯H/H⋯C (11.2%), Cl⋯C/C⋯Cl (7.8%), Cl⋯N/N⋯Cl (3.5%), Cl⋯Cl (1.4%), Co⋯H (1.0%) and C⋯C (0.5%).

## 4. Database survey

A search of the Cambridge Structural Database (CSD, Version 5.44. last update Jun 2023; Groom *et al.*, 2016 ▸) for the 6,6′-dimethyl-2,2′-bipyridinium ion yielded six entries, *viz*. CSD refcodes IQUREU (Yoshikawa, 2021 ▸), IQUREU01 (Yoshikawa *et al.*, 2022 ▸), KARRAA (Jurowska *et al.*, 2021 ▸), QUJVUO (Thangavelu *et al.*, 2015 ▸), UWUKAZ02 (Kobayashi *et al.*, 2014 ▸) and YABGIS (Chan & Baird, 2004 ▸). The mean dihedral angle between the pyridine rings of the DMB^2+^ cations in these structures is 38.75 (10)°

## 5. Synthesis and crystallization

The compound of inter­est was synthesized by a literature method (Jagadeesan *et al.*, 2013 ▸) by dissolving 2.00 mmol (0.3682 g) of the ligand in methanol and adding directly 1.00 mmol (0.1289 g) of anhydrous cobaltous chloride. The whole mixture was refluxed for about an hour. A dark-brown solution was obtained. Afterwards, a sufficient amount of chlorine gas was passed through the solution until precipitation occurred. The precipitate was dissolved in aqueous HCl (0.001 M) by warming to 333 K for 30 min and the resulting mixture was kept undisturbed overnight. The resulting precipitate was discarded and the filtrate was kept for a few weeks until dark-blue crystals of (**I**) appeared (0.067 g).

## 6. Refinement

Crystal data, data collection and structure refinement details are summarized in Table 2 ▸. The H atoms were positioned geometrically (C—H = 0.93–0.96 Å) and were refined using a riding model with *U*_iso_(H) = 1.2*U*_eq_(C).

## Supplementary Material

Crystal structure: contains datablock(s) I. DOI: 10.1107/S2056989024005152/hb8095sup1.cif

Structure factors: contains datablock(s) I. DOI: 10.1107/S2056989024005152/hb8095Isup2.hkl

CCDC reference: 2347766

Additional supporting information:  crystallographic information; 3D view; checkCIF report

## Figures and Tables

**Table 1:** Hydrogen-bond geometry (Å, °)

*D*—H⋯*A*	*D*—H	H⋯*A*	*D*⋯*A*	*D*—H⋯*A*
N1—H1⋯Cl1	0.93 (2)	2.32 (2)	3.2205 (16)	164.5 (18)
N2—H2⋯Cl4^i^	0.87 (2)	2.38 (2)	3.2436 (16)	171 (2)
C4—H4⋯Cl3^ii^	0.93	2.68	3.571 (2)	161
C9—H9⋯Cl2^iii^	0.93	2.79	3.565 (2)	141

Symmetry codes: (i) 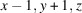; (ii) 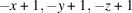; (iii) 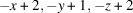.

**Table 2:** Experimental details

Crystal data	
Chemical formula	(C_12_H_14_N_2_)[CoCl_4_]
*M*_r_	386.98
Crystal system, space group	Triclinic, *P* 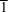
Temperature (K)	301
*a*, *b*, *c* (Å)	6.6419 (16), 7.6512 (19), 15.837 (4)
α, β, γ (°)	99.458 (6), 98.020 (6), 97.046 (6)
*V* (Å^3^)	777.3 (3)
*Z*	2
Radiation type	Mo *K*α
μ (mm^−1^)	1.78
Crystal size (mm)	0.21 × 0.11 × 0.04

Data collection	
Diffractometer	Bruker APEXII CCD
Absorption correction	Multi-scan (*SADABS*; Krause *et al.*, 2015 ▸)
*T*_min_, *T*_max_	0.625, 0.746
No. of measured, independent and observed [*I* > 2σ(*I*)] reflections	13285, 3890, 3661
*R*_int_	0.041
(sin θ/λ)_max_ (Å^−1^)	0.673

Refinement	
*R*[*F*^2^ > 2σ(*F*^2^)], *wR*(*F*^2^), *S*	0.029, 0.081, 1.03
No. of reflections	3890
No. of parameters	180
H-atom treatment	H atoms treated by a mixture of independent and constrained refinement
Δρ_max_, Δρ_min_ (e Å^−3^)	0.41, −0.36

Computer programs: *APEX4* and *SAINT* (Bruker, 2021 ▸), *SHELXT2019/1* (Sheldrick, 2015*a* ▸), *SHELXL2019/2* (Sheldrick, 2015*b* ▸), *PLATON* (Spek, 2020 ▸) and *publCIF* (Westrip, 2010 ▸).

## supplementary crystallographic information

### 6,6'-Dimethyl-2,2'-bipyridine-1,1'-diium tetrachloridocobaltate(II) Crystal data

**Table:**

(C_12_H_14_N_2_)[CoCl_4_]	*Z* = 2
*M**_r_* = 386.98	*F*(000) = 390
Triclinic, *P*1	*D*_x_ = 1.653 Mg m^−^^3^
*a* = 6.6419 (16) Å	Mo *K*α radiation, λ = 0.71073 Å
*b* = 7.6512 (19) Å	Cell parameters from 9272 reflections
*c* = 15.837 (4) Å	θ = 2.7–28.6°
α = 99.458 (6)°	µ = 1.78 mm^−^^1^
β = 98.020 (6)°	*T* = 301 K
γ = 97.046 (6)°	Block, blue
*V* = 777.3 (3) Å^3^	0.21 × 0.11 × 0.04 mm

### 6,6'-Dimethyl-2,2'-bipyridine-1,1'-diium tetrachloridocobaltate(II) Data collection

**Table:**

Bruker APEXII CCD diffractometer	3661 reflections with *I* > 2σ(*I*)
φ and ω scans	*R*_int_ = 0.041
Absorption correction: multi-scan (SADABS; Krause *et al.*, 2015)	θ_max_ = 28.6°, θ_min_ = 2.7°
*T*_min_ = 0.625, *T*_max_ = 0.746	*h* = −8→8
13285 measured reflections	*k* = −10→10
3890 independent reflections	*l* = −21→21

### 6,6'-Dimethyl-2,2'-bipyridine-1,1'-diium tetrachloridocobaltate(II) Refinement

**Table:**

Refinement on *F*^2^	Primary atom site location: dual
Least-squares matrix: full	Hydrogen site location: mixed
*R*[*F*^2^ > 2σ(*F*^2^)] = 0.029	H atoms treated by a mixture of independent and constrained refinement
*wR*(*F*^2^) = 0.081	*w* = 1/[σ^2^(*F*_o_^2^) + (0.0415*P*)^2^ + 0.2392*P*] where *P* = (*F*_o_^2^ + 2*F*_c_^2^)/3
*S* = 1.03	(Δ/σ)_max_ = 0.002
3890 reflections	Δρ_max_ = 0.41 e Å^−^^3^
180 parameters	Δρ_min_ = −0.36 e Å^−^^3^
0 restraints	

### 6,6'-Dimethyl-2,2'-bipyridine-1,1'-diium tetrachloridocobaltate(II) Special details

**Table:**

Geometry. All esds (except the esd in the dihedral angle between two l.s. planes) are estimated using the full covariance matrix. The cell esds are taken into account individually in the estimation of esds in distances, angles and torsion angles; correlations between esds in cell parameters are only used when they are defined by crystal symmetry. An approximate (isotropic) treatment of cell esds is used for estimating esds involving l.s. planes.

### 6,6'-Dimethyl-2,2'-bipyridine-1,1'-diium tetrachloridocobaltate(II) Fractional atomic coordinates and isotropic or equivalent isotropic displacement parameters (Å^2^)

**Table:**

	*x*	*y*	*z*	*U*_iso_*/*U*_eq_	
Co1	0.83193 (3)	0.11776 (3)	0.75825 (2)	0.02835 (8)	
Cl1	0.50544 (6)	0.08040 (5)	0.79190 (3)	0.03681 (10)	
Cl2	1.04301 (7)	0.30332 (6)	0.87001 (3)	0.04763 (12)	
Cl3	0.82188 (7)	0.26717 (7)	0.64540 (3)	0.04459 (11)	
Cl4	0.91033 (8)	−0.16382 (6)	0.71718 (3)	0.04905 (12)	
N1	0.34377 (19)	0.34550 (17)	0.66800 (8)	0.0288 (2)	
H1	0.371 (3)	0.276 (3)	0.7098 (14)	0.043*	
N2	0.2849 (2)	0.69414 (16)	0.82797 (8)	0.0295 (2)	
H2	0.178 (3)	0.719 (3)	0.7963 (14)	0.044*	
C1	0.2787 (3)	0.0670 (3)	0.56372 (13)	0.0511 (5)	
H1A	0.235167	0.025817	0.502509	0.077*	
H1B	0.181069	0.013236	0.594770	0.077*	
H1C	0.411174	0.033861	0.580711	0.077*	
C2	0.2923 (2)	0.2659 (2)	0.58417 (10)	0.0351 (3)	
C3	0.2537 (3)	0.3744 (3)	0.52323 (11)	0.0432 (4)	
H3	0.220294	0.323926	0.464580	0.052*	
C4	0.2648 (3)	0.5561 (3)	0.54950 (11)	0.0465 (4)	
H4	0.239832	0.628226	0.508449	0.056*	
C5	0.3130 (3)	0.6328 (2)	0.63697 (11)	0.0386 (3)	
H5	0.317851	0.755367	0.655242	0.046*	
C6	0.3535 (2)	0.52315 (19)	0.69586 (9)	0.0276 (3)	
C7	0.4097 (2)	0.58981 (18)	0.79017 (9)	0.0276 (3)	
C8	0.5805 (3)	0.5515 (2)	0.83936 (11)	0.0360 (3)	
H8	0.669290	0.481996	0.814006	0.043*	
C9	0.6170 (3)	0.6193 (2)	0.92811 (11)	0.0440 (4)	
H9	0.731908	0.595592	0.962668	0.053*	
C10	0.4840 (3)	0.7213 (2)	0.96494 (10)	0.0441 (4)	
H10	0.507794	0.764127	1.024450	0.053*	
C11	0.3147 (3)	0.7606 (2)	0.91364 (10)	0.0368 (3)	
C12	0.1623 (4)	0.8715 (3)	0.94657 (14)	0.0555 (5)	
H12A	0.186823	0.894239	1.008855	0.083*	
H12B	0.025769	0.808301	0.925793	0.083*	
H12C	0.176477	0.983126	0.926248	0.083*	

### 6,6'-Dimethyl-2,2'-bipyridine-1,1'-diium tetrachloridocobaltate(II) Atomic displacement parameters (Å^2^)

**Table:**

	*U*^11^	*U*^22^	*U*^33^	*U*^12^	*U*^13^	*U*^23^
Co1	0.02780 (12)	0.03196 (12)	0.02530 (12)	0.00566 (8)	0.00268 (8)	0.00579 (8)
Cl1	0.03370 (19)	0.03675 (19)	0.0430 (2)	0.00376 (14)	0.01266 (15)	0.01194 (15)
Cl2	0.0492 (2)	0.0456 (2)	0.0389 (2)	−0.00486 (18)	−0.01213 (18)	0.00607 (17)
Cl3	0.0398 (2)	0.0659 (3)	0.0347 (2)	0.00992 (19)	0.00815 (16)	0.02524 (19)
Cl4	0.0507 (3)	0.0430 (2)	0.0507 (3)	0.02070 (19)	0.00004 (19)	−0.00242 (18)
N1	0.0270 (6)	0.0315 (6)	0.0266 (6)	0.0068 (5)	0.0002 (4)	0.0032 (5)
N2	0.0350 (6)	0.0275 (6)	0.0265 (6)	0.0043 (5)	0.0051 (5)	0.0068 (4)
C1	0.0499 (10)	0.0451 (10)	0.0481 (10)	0.0075 (8)	0.0000 (8)	−0.0142 (8)
C2	0.0264 (7)	0.0447 (8)	0.0298 (7)	0.0061 (6)	0.0014 (5)	−0.0034 (6)
C3	0.0377 (8)	0.0659 (11)	0.0241 (7)	0.0080 (8)	0.0028 (6)	0.0040 (7)
C4	0.0482 (10)	0.0668 (12)	0.0301 (8)	0.0134 (9)	0.0054 (7)	0.0224 (8)
C5	0.0462 (9)	0.0390 (8)	0.0343 (8)	0.0109 (7)	0.0058 (7)	0.0149 (6)
C6	0.0266 (6)	0.0315 (7)	0.0253 (6)	0.0063 (5)	0.0031 (5)	0.0062 (5)
C7	0.0312 (7)	0.0250 (6)	0.0257 (6)	0.0018 (5)	0.0020 (5)	0.0061 (5)
C8	0.0353 (8)	0.0330 (7)	0.0371 (8)	0.0045 (6)	−0.0039 (6)	0.0078 (6)
C9	0.0470 (9)	0.0429 (9)	0.0355 (8)	−0.0034 (7)	−0.0117 (7)	0.0117 (7)
C10	0.0619 (11)	0.0393 (8)	0.0247 (7)	−0.0076 (8)	0.0003 (7)	0.0045 (6)
C11	0.0511 (9)	0.0294 (7)	0.0298 (7)	−0.0015 (6)	0.0123 (7)	0.0058 (6)
C12	0.0712 (14)	0.0506 (11)	0.0466 (10)	0.0094 (10)	0.0273 (10)	−0.0002 (8)

### 6,6'-Dimethyl-2,2'-bipyridine-1,1'-diium tetrachloridocobaltate(II) Geometric parameters (Å, º)

**Table:**

Co1—Cl2	2.2600 (6)	C4—C5	1.389 (2)
Co1—Cl3	2.2720 (6)	C4—H4	0.9300
Co1—Cl4	2.2901 (7)	C5—C6	1.374 (2)
Co1—Cl1	2.2997 (7)	C5—H5	0.9300
N1—C2	1.3441 (19)	C6—C7	1.4758 (19)
N1—C6	1.3490 (19)	C7—C8	1.375 (2)
N1—H1	0.93 (2)	C8—C9	1.392 (2)
N2—C11	1.346 (2)	C8—H8	0.9300
N2—C7	1.3511 (19)	C9—C10	1.376 (3)
N2—H2	0.87 (2)	C9—H9	0.9300
C1—C2	1.492 (3)	C10—C11	1.387 (3)
C1—H1A	0.9600	C10—H10	0.9300
C1—H1B	0.9600	C11—C12	1.492 (3)
C1—H1C	0.9600	C12—H12A	0.9600
C2—C3	1.390 (3)	C12—H12B	0.9600
C3—C4	1.374 (3)	C12—H12C	0.9600
C3—H3	0.9300		
			
Cl2—Co1—Cl3	105.46 (3)	C6—C5—C4	118.36 (16)
Cl2—Co1—Cl4	117.91 (2)	C6—C5—H5	120.8
Cl3—Co1—Cl4	110.39 (2)	C4—C5—H5	120.8
Cl2—Co1—Cl1	108.93 (2)	N1—C6—C5	119.83 (14)
Cl3—Co1—Cl1	107.287 (19)	N1—C6—C7	116.98 (12)
Cl4—Co1—Cl1	106.46 (2)	C5—C6—C7	123.19 (14)
C2—N1—C6	123.47 (14)	N2—C7—C8	119.92 (14)
C2—N1—H1	119.4 (14)	N2—C7—C6	116.93 (13)
C6—N1—H1	117.1 (14)	C8—C7—C6	123.15 (14)
C11—N2—C7	123.32 (14)	C7—C8—C9	118.30 (16)
C11—N2—H2	117.4 (14)	C7—C8—H8	120.9
C7—N2—H2	119.2 (14)	C9—C8—H8	120.9
C2—C1—H1A	109.5	C10—C9—C8	120.32 (16)
C2—C1—H1B	109.5	C10—C9—H9	119.8
H1A—C1—H1B	109.5	C8—C9—H9	119.8
C2—C1—H1C	109.5	C9—C10—C11	120.25 (15)
H1A—C1—H1C	109.5	C9—C10—H10	119.9
H1B—C1—H1C	109.5	C11—C10—H10	119.9
N1—C2—C3	117.74 (15)	N2—C11—C10	117.86 (16)
N1—C2—C1	117.33 (16)	N2—C11—C12	117.45 (17)
C3—C2—C1	124.93 (16)	C10—C11—C12	124.69 (17)
C4—C3—C2	120.12 (15)	C11—C12—H12A	109.5
C4—C3—H3	119.9	C11—C12—H12B	109.5
C2—C3—H3	119.9	H12A—C12—H12B	109.5
C3—C4—C5	120.43 (16)	C11—C12—H12C	109.5
C3—C4—H4	119.8	H12A—C12—H12C	109.5
C5—C4—H4	119.8	H12B—C12—H12C	109.5
			
C6—N1—C2—C3	2.1 (2)	N1—C6—C7—N2	−128.78 (14)
C6—N1—C2—C1	−177.24 (15)	C5—C6—C7—N2	51.6 (2)
N1—C2—C3—C4	−1.2 (3)	N1—C6—C7—C8	51.7 (2)
C1—C2—C3—C4	178.05 (18)	C5—C6—C7—C8	−127.95 (17)
C2—C3—C4—C5	−0.5 (3)	N2—C7—C8—C9	1.4 (2)
C3—C4—C5—C6	1.4 (3)	C6—C7—C8—C9	−179.06 (15)
C2—N1—C6—C5	−1.2 (2)	C7—C8—C9—C10	0.3 (3)
C2—N1—C6—C7	179.19 (14)	C8—C9—C10—C11	−1.4 (3)
C4—C5—C6—N1	−0.6 (2)	C7—N2—C11—C10	1.0 (2)
C4—C5—C6—C7	178.99 (15)	C7—N2—C11—C12	−179.01 (15)
C11—N2—C7—C8	−2.1 (2)	C9—C10—C11—N2	0.8 (2)
C11—N2—C7—C6	178.36 (14)	C9—C10—C11—C12	−179.26 (18)

### 6,6'-Dimethyl-2,2'-bipyridine-1,1'-diium tetrachloridocobaltate(II) Hydrogen-bond geometry (Å, º)

**Table:**

*D*—H···*A*	*D*—H	H···*A*	*D*···*A*	*D*—H···*A*
N1—H1···Cl1	0.93 (2)	2.32 (2)	3.2205 (16)	164.5 (18)
N2—H2···Cl4^i^	0.87 (2)	2.38 (2)	3.2436 (16)	171 (2)
C4—H4···Cl3^ii^	0.93	2.68	3.571 (2)	161
C9—H9···Cl2^iii^	0.93	2.79	3.565 (2)	141

Symmetry codes: (i) *x*−1, *y*+1, *z*; (ii) −*x*+1, −*y*+1, −*z*+1; (iii) −*x*+2, −*y*+1, −*z*+2.
